# AURKA inhibition induces Ewing’s sarcoma apoptosis and ferroptosis through NPM1/YAP1 axis

**DOI:** 10.1038/s41419-024-06485-0

**Published:** 2024-01-29

**Authors:** Huimou Chen, Jing Hu, Xilin Xiong, Hongling Chen, Biaojun Lin, Yusong Chen, Yang Li, Di Cheng, Zhihua Li

**Affiliations:** 1 Department of Oncology, Sun Yat-sen Memorial Hospital of Sun Yat-sen University, Guangzhou, China; 2 Department of Clinical Laboratory, The Six Affiliated Hospital of Sun Yat-sen University, Guangzhou, China; 3 Biomedical Innovation Center, The Sixth Affiliated Hospital, Sun Yat-sen University, Guangzhou, China; 4 Department of Oncology, Medical Centre of Pediatric, Sun Yat-sen Memorial Hospital, Sun Yat-sen University, Guangzhou, China; 5 Department Of Clinical Laboratory, Maoming People’s Hospital, Maoming, Guangdong People’s Republic of China

**Keywords:** Targeted therapies, Paediatric cancer

## Abstract

Ewing’s sarcoma (ES) is a rare and highly aggressive malignant tumor arising from bone and soft tissue. Suffering from intractable or recurrent diseases, the patients’ therapy options are very limited. It is extremely urgent to identify novel potential therapeutic targets for ES and put them into use in clinical settings. In the present study, high-throughput screening of a small molecular pharmacy library was performed. The killing effect of the Aurora kinase A (AURKA) inhibitor TCS7010 in ES cells was identified, and AURKA was selected as the research object for further study. Disparate suppressants were adopted to study the cell death manner of TCS7010. TCS7010 and RNA silencing were used to evaluate the functions of AURKA in the apoptosis and ferroptosis of ES cells. Co-immunoprecipitation assay was used to investigate the correlation of AURKA and nucleophosmin1 (NPM1) in ES. Nude-mice transplanted tumor model was used for investigating the role of AURKA in ES in vivo. Investigations into the protein activities of AURKA were conducted using ES cell lines and xenograft models. AURKA was found to be prominently upregulated in ES. The AURKA expression level was remarkably connected to ES patients’ shorter overall survival (OS) and event-free survival (EFS). Furthermore, AURKA inhibition markedly induced the apoptosis and ferroptosis of ES cells and attenuated tumorigenesis in vivo. On the part of potential mechanisms, it was found that AURKA inhibition triggered the apoptosis and ferroptosis of ES cells through the NPM1/Yes1 associated transcriptional regulator (YAP1) axis, which provides new insights into the tumorigenesis of ES. AURKA may be a prospective target for clinical intervention in ES patients.

## Introduction

Ewing’s sarcoma (ES) is a malignant round-cell tumor originating from the bone or soft tissue, which predominately affects children, adolescents and young adults, with an estimated incidence rate of 1.5 cases per million globally, and slightly more male cases than female cases [1]. With the recent advances in imaging technologies, surgical techniques, radiotherapy, and chemotherapy, the 5-year survival rate of patients with ES has reached 60–70% [2]. However, ES is related to aggressive behaviors with a high propensity for early-onset dissemination, and existing therapeutic approaches are unsuccessful against metastatic and recurrent disorders. About 25% of ES patients are diagnosed with metastatic disease, and patients with refractory or recurrent disease have poor outcomes, with a 5-year survival rate lower than 30% [3]. ES is characterized by a variety of fusions involving the Ewing sarcoma breakpoint region 1 (EWSR1) gene and E26 transformation-specific (ETS) transcription factors, with EWSR1-FLI1 being the most common fusion subtype. The EWSR1-FLI1 fusion protein behaves as an aberrant transcription factor, which modulates the expressions of specific targets of EWSR1 [1]. However, the development of new molecular compounds that can directly target fusion proteins has always been a daunting challenge [4]. Therefore, finding new and powerful therapeutic options for ES requires elucidating other potential molecular pathways driving ES development.

Acquired or inherent resistance to cell apoptosis is the main cause of chemotherapy failure [5]. This emphasizes how critical it is to create methods of conquering apoptosis resistance or inducing non-apoptotic forms of programmed cell death in ES. One such non-apoptotic modality of cell death, ferroptosis, is caused by massive lipid peroxidation-mediated membrane damage [6, 7]. In recent years, increasing research revealed that ferroptosis is implicated in various human diseases, including stroke, intracerebral hemorrhage, kidney degeneration and cancer [8]. Numerous cancer cells, such as breast cancer, non-small cell lung cancer (NSCLC), liver cancer, and rhabdomyosarcoma, have been found to be susceptible to ferroptosis [9–12]. Ferroptosis inducers significantly reduce tumor growth and improve the sensitivity of chemotherapeutic drugs [13]. Therefore, inducing ferroptosis has become a novel potential strategy for cancer therapy.

Aurora kinase A (AURKA) is a member of the highly homologous family of serine/threonine kinases, which is recognized to be essential in controlling cell cycle and division [14]. Meanwhile, AURKA was reported to act as an oncogene in a variety of cancers [15, 16]. However, the expression level and the role of AURKA in ES are still poorly understood. In this study, small-molecule library screening was performed to identify compounds impairing ES cell growth, and it was found that TCS7010 exhibited considerable anticancer activity in human ES via selectively inhibiting AURKA and inducting apoptosis and ferroptosis.

Besides, AURKA was significantly elevated in ES, which repressed the apoptosis and ferroptosis in ES, and was correlated with poor survival. Thus, our study shed light on the utilization of AURKA as a relevant therapeutic target for patients with ES.

## Results

### Anticancer activity of TCS7010 in ES cell lines

**Fig. 1:**
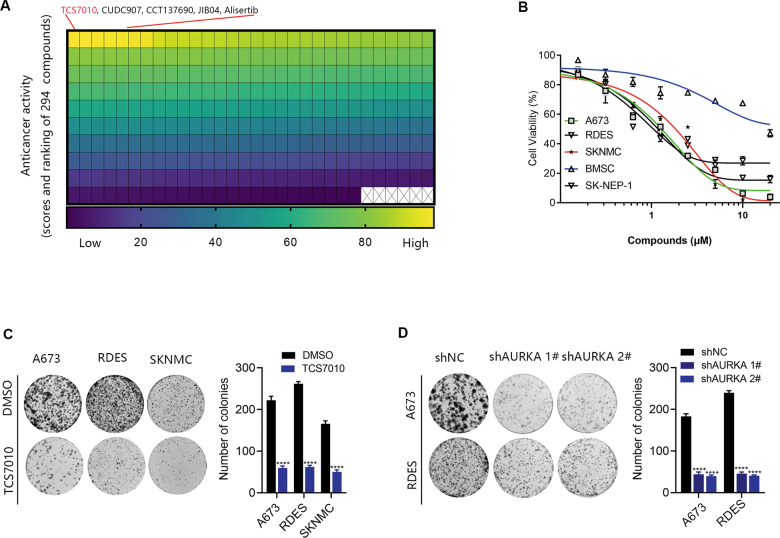
TCS7010 was identified as an effective anticancer agent in ES cells. **A** Effective drugs based on high-throughput screening of the small molecule pharmacy library containing 294 compounds in A673. **B** BMSC was used as the normal control. Indicated ES or normal BMSC cells were treated with TCS7010 for 48 h, and then cell viability was assayed by CCK-8. **C** Colony formation assay indicated that after treated A673, RDES and SKNMC with 1 μM TCS7010, colony formation ability was obviously decreased compared to the DMSO control group. **D** Cell proliferation ability was evaluated with colony formation assay after AURKA was knocked down with shRNA. Values represented the mean ± SD from 3 independent experiments. ****p < 0.0001.

We used ES cell lines A673 to screen compounds with anticancer activity against ES from a commercially available small-molecule library containing 294 compounds. In the primary cytotoxicity assays using a single concentration (2 μM), we identified the following top 5 compounds: TCS7010 (an AURKA inhibitor), CUDC907 (a dual HDAC and PI3K inhibitor), CCT137690 (an aurora kinase inhibitor), JIB04 (a dual HDAC and PI3K inhibitor), and Alisertib (an AURKA inhibitor) (Fig. 1A). The top 5 compounds contained 2 AURKA inhibitors, we therefore focused on the study of TCS7010 for the following experiments due to its previously unidentified role in ES. In addition to A673, TCS7010 killed other human ES cell lines in a dose-dependent manner, including SKNMC, RDES and SK-NEP-1. By comparison, normal BMSC cells were resistant to TCS7010 (Fig. 1B). Colony formation assays confirmed that the TCS7010 therapy or AURKA knockdown severely impaired the reproductive integrity of the ES cells (Fig. 1C, D). These findings collectively imply that TCS7010 has anticancer activity in human ES cells.

### AURKA was upregulated in ES and associated with poor prognosis of patients

**Fig. 2:**
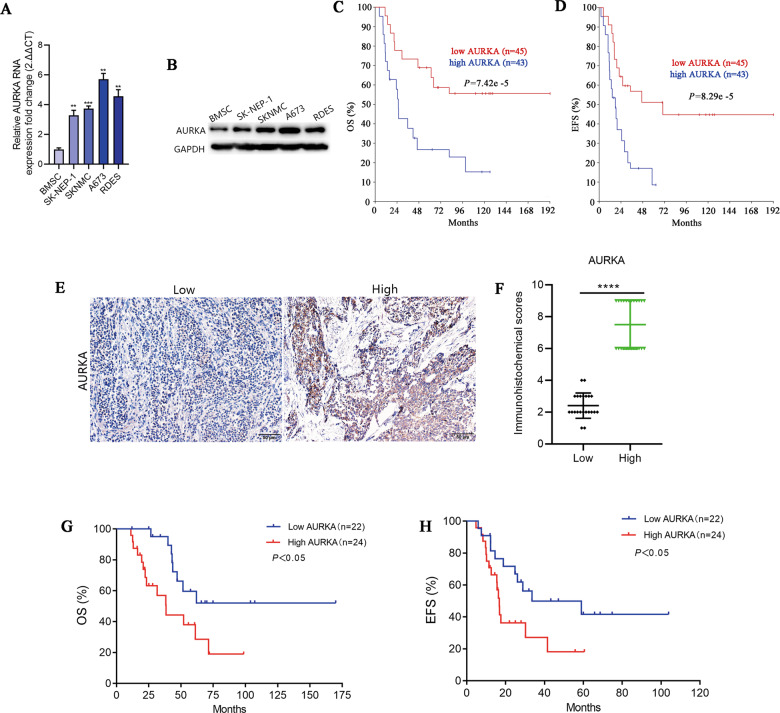
AURKA was upregulated and negatively correlated with the prognosis of ES. **A** RT-qPCR analysis revealed the mRNA expression level of AURKA in BMSC and 4 ES cell lines. **B** WB analysis revealed the protein expression level of AURKA in BMSC and 4 ES cell lines. The corresponding original western blots are shown in Fig. S7. **C**, **D** K-M survival curve of the relationship between AURKA expression level and OS/EFS in ES patients based on the online R2 genomics analysis platform. **E** Representative images of the expression of AURKA protein in patients with ES detected by IHC (×200). **F** The quantitative analysis of AURKA immunohistochemical staining. **G**, **H** OS and EFS curves of ES patients expressing high-level or low-level of AURKA. Values represented the mean ± SD from 3 independent experiments. **p < 0.01, ***p < 0.001, ****p < 0.0001.

To further confirm the expression of AURKA in ES, RT-qPCR and WB were performed in ES cell lines. As expected, compared with BMSC, AURKA was significantly upregulated in ES cell lines (Fig. 2A, B). We investigated the association between AURKA expression and survival in human ES (*n* = 88) cohorts from the online R2 genomics analysis and visualization platform. We found that high expression of AURKA was notably correlated with shorter OS (*p* < 0.0001) and EFS (*p* < 0.0001) in ES patients (Fig. 2C, D). Expression levels of AURKA in ES tissues were evaluated with IHC staining, and the representative images were showed in Fig. 2E, and the quantitative analysis was showed in Fig. 2F. Accordingly, the survival analysis of follow-up data showed that high expression of AURKA was notably correlated with shorter OS (*p* < 0.05) and EFS (*p* < 0.05) in ES patients at our center (Fig. 2G, H).

### TCS7010 induced the apoptosis and ferroptosis of ES cells

**Fig. 3:**
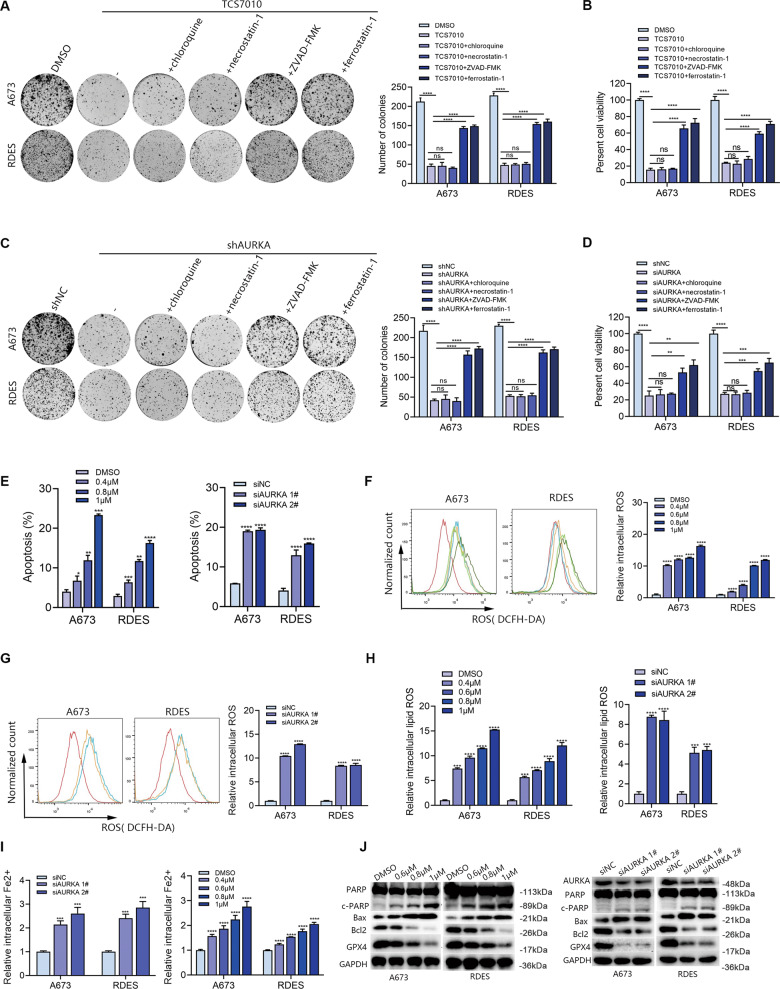
AURKA inhibition induced the apoptosis and ferroptosis in ES cells. **A** Colony formation assay determined the reproductive ability of ES cells treated with TCS7010 (1 μM) in the absence or presence of indicated cell death inhibitors (10 µM ZVAD-FMK, 10 µM necrostatin-1, 10 µM ferrostatin-1 and 50 µM chloroquine). **B** A673 and RDES cells were treated with TCS7010 (1 μM) in the absence or presence of indicated cell death inhibitors (10 µM ZVAD-FMK, 10 µM necrostatin-1, 10 µM ferrostatin-1 and 50 µM chloroquine) for 48 h. Cell viability was assayed with CCK-8. **C** Colony formation assay determined the reproductive ability of ES cells with AURKA knockdown in the absence or presence of indicated cell death inhibitors (10 µM ZVAD-FMK, 10 µM necrostatin-1, 10 µM ferrostatin-1 and 50 µM chloroquine). **D** A673 and RDES cells with AURKA knockdown in the absence or presence of indicated cell death inhibitors (10 µM ZVAD-FMK, 10 µM necrostatin-1, 10 µM ferrostatin-1 and 50 µM chloroquine) for 48 h. Cell viability was assayed with CCK-8. **E** Columnar statistical chart indicated changes in the apoptosis rates of ES cell lines after AURKA inhibition with TCS7010 or siRNA. **F**, **G** Detection of the intracellular ROS levels in ES cells after TCS7010 treatment or AURKA knockdown. **H** Detection of the intracellular lipid ROS levels in ES cells after TCS7010 treatment or AURKA knockdown. **I** Changes of the relative intracellular Fe2+ levels in ES cells after AURKA inhibition with TCS7010 or siRNA. **J** WB analysis indicated changes of the apoptosis-related gene markers (PARP, Bcl2, Bax) and the ferroptosis-related marker GPX4 after AURKA inhibition in ES cells. The corresponding original western blots were showed in Fig. S8. Values represented the mean ± SD from 3 independent experiments. ns non significance, *p < 0.05, **p < 0.01, ***p < 0.001, ****p < 0.0001.

To reveal the mechanism of TCS7010, the death mode was analyzed. The apoptosis inhibitor ZVAD-FMK, cell necrosis inhibitor necrostatin-1, cell autophagy inhibitor chloroquine and ferroptosis inhibitor ferrostatin-1 were used. The results showed that there existed no remarkable variation in the cell viability after the combination of TCS7010 with necrostatin-1 or chloroquine, indicating that TCS7010 cannot induce cell necrosis or cell autophagy in ES. However, the effect of TCS7010 was partially inhibited by ZVAD-FMK or ferrostatin-1(Fig. 3A, B). Consistently, ZVAD-FMK or ferrostatin-1 partially prevented the effect of AURKA knockdown (Fig. 3C, D). Taken together, cell apoptosis and ferroptosis are partially induced to achieve the impact of TCS7010 or AURKA knockdown.

We then investigated the role of TCS7010 in inducing apoptosis in ES cells. The results of flow cytometry revealed that TCS7010 significantly induced ES cell apoptosis in a dose-dependent manner. Consistently, AURKA knockdown significantly increased apoptosis in ES cells (Fig. 3E). The function and mechanism of TCS7010’s induction of ferroptosis in ES cells were then investigated. After treatment with TCS7010, there was a dose-dependent increase in the accumulation of intracellular ROS, lipid ROS, and Fe2 + (Fig. 3F–I). According to the WB results, after AURKA inhibition with TCS7010 or genetic knockdown, the relative levels of anti-apoptotic protein Bcl2 were decreased in a dose-dependent manner. AURKA inhibition also greatly boosted the apoptotic proteins Bax and c-PARP, while the protein level of GPX4 was prominently reduced after AURKA inhibition (Fig. 3J). These results suggested that TCS7010 and AURKA knockdown can induce the apoptosis and ferroptosis of ES cells.

As a cell cycle-related protein, AURKA exerts crucial effects in cell cycle regulation [17]. The results of our study also demonstrated that AURKA could regulate the cell cycle process of ES cells since significant G2/M phrase arrest was observed after AURKA inhibition (Fig. S1A). Given that AURKA is essential for cell cycle progression, we next explored whether impaired cell cycle regulation is required for AURKA-mediated apoptosis and ferroptosis inhibition in ES cells. We then tested the capacity of other cell cycle inhibitors other than TCS7010 to induce apoptosis and ferroptosis. Vincristine (VCR), a first-line chemotherapy agent for ES [18, 19], can induce cancer cell death, as they induce cell cycle arrest in the G2/M phase (Fig. S1B). However, ferrostatin-1 had no effects on cell death induced by VCR (Fig. S1C). These findings indicate that cell cycle arrest may not be essential for ferroptosis induction in ES cells following AURKA inhibition. In contrast, ZVAD-FMK partly prevented cell death induced by VCR, which revealed that cell cycle inhibitor affects the apoptosis to a certain degree, indicating the involvement of apoptosis induction of AURKA when regulating the cell cycle procedure.

### AURKA exerted a pro-tumor effect in vivo

**Fig. 4:**
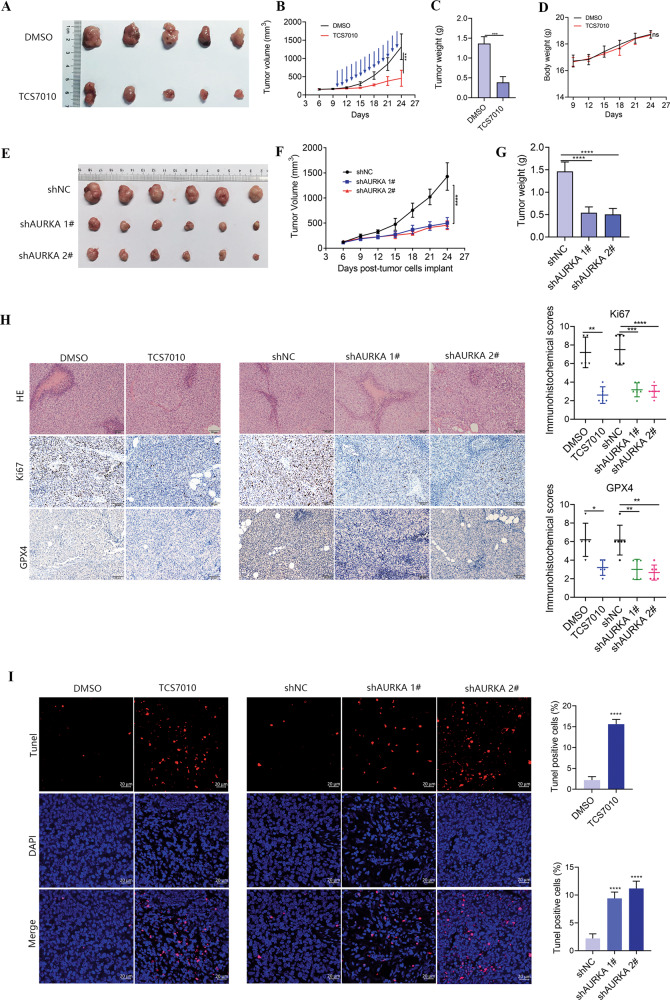
AURKA inhibition significantly induced ES apoptosis and ferroptosis in vivo. **A** Representative photographs of isolated tumors at day 14 after treatment. **B** Tumor growth curves of the xenograft tumors. Blue arrows indicated the exact days for TCS7010 administration. **C** Box plot of xenograft tumor weight between the control and TCS7010 treatment groups. **D** Mice weight curves during the experiment. **E** Representative photographs of isolated tumors from shNC and shAURKA groups. **F** Tumor growth curves of the xenograft tumors from shNC and shAURKA groups. **G** Box plot of xenograft tumor weight between shNC and shAURKA groups. **H** Representative IHC images of Ki67 and GPX4 in xenografts from the DMSO control, TCS7010 treatment, shNC, and shAURKA groups at the end of the experiment. **I** Representative confocal images of Tunel in xenografts from the DMSO control, TCS7010 treatment, shNC, and shAURKA groups at the end of the experiment. ns non-significance, *p < 0.05, **p < 0.01, ***p < 0.001, ****p < 0.0001.

In order to deeply validate the anti-tumor effect of AURKA inhibitor (TCS7010) in vivo, we established a xenograft model in mice with A673 cells. When the tumor volume reached 80–100 mm^3^, mice were stochastically divided into vehicle control group and TCS7010 treatment group. TCS7010 was injected intraperitoneally at a dosage of 40 mg/kg/d for 14 days. To further verify the pro-tumor effect of AURKA in vivo, a xenograft model was established in mice with A673 cells with AURKA knockdown (shAURKA) or not (shNC). As expected, AURKA inhibitor TCS7010 inhibited ES growth in vivo (Fig. 4A–C). Moreover, the mice body weights were comparable between the two groups during the experiment, indicating that minimal adverse effects were caused by TCS7010 (Fig. 4D). Consistently, the tumor growth rate in the shNC group was significantly higher than that in the shAURKA group (Fig. 4E–G). IHC staining also exhibited that tumors treated by TCS7010 or AURKA knockdown had fewer Ki67-positive cells and lower GPX4 expression (Fig. 4H). In addition, Tunel assays were performed and showed that TCS7010 and AURKA knockdown markedly elevated the number of Tunel-positive apoptotic cells (Fig. 4I). To sum up, AURKA exerted a pro-tumor effect in vivo. AURKA is identified as a new potential therapeutic target in ES.

### AURKA phosphorylated NPM1 at threonine 95 (Thr95) to promote ES apoptosis and ferroptosis resistance

**Fig. 5:**
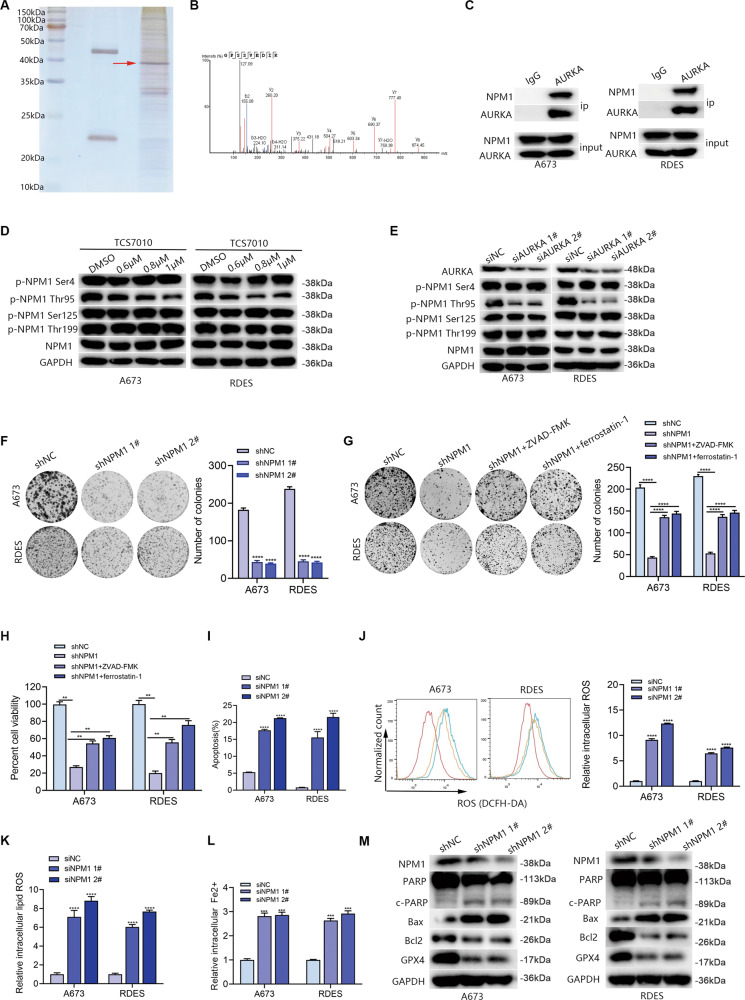
AURKA promoted ES cell apoptosis and ferroptosis resistance via phosphorylating Thr95 of NPM1. **A** Silver staining of co-immunoprecipitation with AURKA antibody in A673. **B** Results of protein mass spectrometry revealed the physical interaction of AURKA and NPM1. **C** Results of immunoprecipitation to confirm the interaction relationship of AURKA and NPM1 in ES cells. The corresponding original western blots are shown in Fig. S9. **D** WB analysis identified the expression level changes of p-NPM1 at Ser4, Thr95, Ser125, Thr199, and the total NPM1 in ES cell lines after treatment with TCS7010. The corresponding original western blots are shown in Fig. S10. **E** WB analysis identified the expression level changes of p-NPM1 at Ser4, Thr95, Ser125, Thr199, and the total NPM1 in ES cell lines after AURKA knockdown. The corresponding original western blots are shown in Fig. S10. **F** Colony formation assay showed the reproductive ability of ES cells with NPM1 knockdown. **G** Colony formation assay determined the reproductive ability of ES cells with NPM1 knockdown in the absence or presence of indicated cell death inhibitors (10 µM ZVAD-FMK and 10 µM ferrostatin-1). **H** Columnar statistical chart based on the CCK-8 assays indicated the cell viability after NPM1 knockdown in the absence or presence of indicated cell death inhibitors (10 µM ZVAD-FMK and 10 µM ferrostatin-1) for 48 h. **I** Columnar statistical chart indicated changes in the apoptosis rates of ES cell lines after NPM1 inhibition with siRNA. **J** Detection of the intracellular ROS levels in ES cells after NPM1 inhibition. **K** Detection of the intracellular lipid ROS levels in ES cells after NPM1 inhibition. **L** Changes of the relative intracellular Fe2+ levels in ES cells after NPM1 inhibition. **M** WB analysis indicated changes in the apoptosis-related gene markers (PARP, Bcl2, Bax) and the ferroptosis-related marker GPX4 after NPM1 inhibition in ES cells. The corresponding original western blots are shown in Fig. S11. Values represented the mean ± SD from 3 independent experiments. **p < 0.01, ***p < 0.001, ****p < 0.0001.

**Fig. 6:**
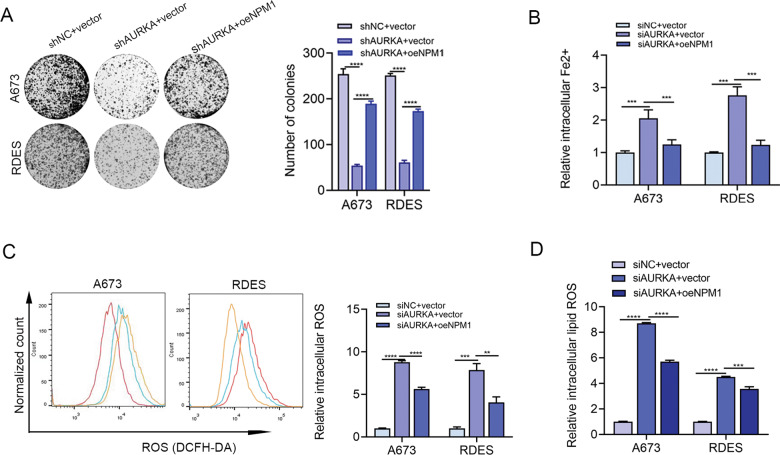
AURKA inhibition induced ES cell apoptosis and ferroptosis through NPM1. **A** Colony formation assay of A673 and RDES after genetic knockdown of AURKA alone or combined with NPM1 overexpression. **B** Detection of intracellular Fe2+ after genetic knockdown of AURKA alone or combined with NPM1 overexpression. **C** Detection of intracellular ROS levels after AURKA knockdown alone or combined with NPM1 overexpression. **D** Detection of intracellular lipid ROS levels after AURKA knockdown alone or combined with NPM1 overexpression. Values represented the mean ± SD from 3 independent experiments. **p < 0.01,  ***p < 0.001, ****p < 0.0001.

The possible molecules that could interact with AURKA in ES cells were next investigated. The results of silver staining indicated that A673 had discrete bands between 35 and 40 kDa that were precipitated by the anti-AURKA antibody (Fig. 5A), which was identified as NPM1 by mass spectrometry (Fig. 5B). Since the high intensity of NPM1 and its potential role in regulating ferroptosis [20, 21], we inferred that AURKA might regulate ferroptosis in ES through NPM1. Bioinformatics analysis (http://www.hitpredict.org/) revealed that AURKA interacted with NPM1 with an interaction coefficient index of 0.571, which was further confirmed by immunoprecipitation (Fig. 5C). These results indicated that AURKA and NPM1 have physical contact. Interestingly, WB results showed that TCS7010 treatment in both A673 and RDES cells brought about an obvious decline in the phosphorylation level of Thr95 of NPM1 (p-NPM1 Thr95) in a dose-dependent manner, while the phosphorylation levels of Ser4, Ser125, Thr199 and the total NPM1 showed no obvious changes (Fig. 5D). Furthermore, knockdown of AURKA significantly decreased p-NPM1 Thr95, while other types of phosphorylated-NPM1 mentioned above appeared to have no obvious changes as expected (Fig. 5E). Knockdown of NPM1 significantly impaired cell proliferation (Fig. 5F), and induced cell apoptosis and ferroptosis (Fig. 5G–M) in ES cells. Moreover, the phosphorylated status of NPM1 Thr95 was important for ES cell growth (Fig. S3). Further functional assays demonstrated that knockdown of AURKA significantly impaired the proliferation and induced ferroptosis of ES cells, while overexpression of NPM1 partly reversed the proliferation arrest and ferroptosis of tumor cells induced by AURKA knockdown (Fig. 6A–D). Altogether, these findings provided strong evidence that AURKA regulates ES cell proliferation, apoptosis and ferroptosis via phosphorylating NPM1 Thr95.

### AURKA promoted YAP1 stability by phosphorylating NPM1

**Fig. 7:**
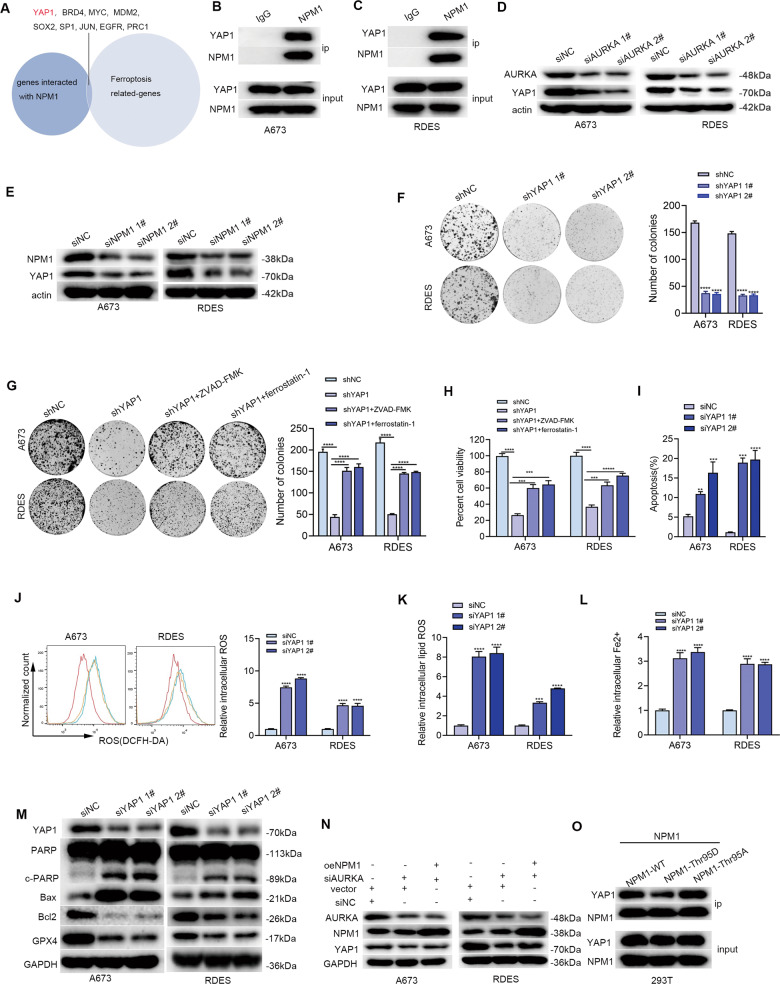
AURKA promoted YAP1 stability by binding NPM1. **A** The overlap of genes interacted with NPM1 and genes associated with ferroptosis contained 9 known oncogenes in ES. **B**, **C** Results of immunoprecipitation to confirm the interaction relationship of NPM1 and YAP1 in ES cells. The corresponding original western blots are shown in Fig. S11. **D**, **E** WB analysis indicated the downregulation of YAP1 after AURKA or NPM1 inhibition in ES cells. The corresponding original western blots are shown in Fig. S12. **F** Colony formation assay showed the reproductive ability of ES cells with YAP1 knockdown. **G** Colony formation assay revealed the reproductive ability of ES cells with NPM1 knockdown in the absence or presence of indicated cell death inhibitors (10 µM ZVAD-FMK and 10 µM ferrostatin-1). **H** Results of CCK-8 assays indicated the cell viability after YAP1 knockdown in the absence or presence of indicated cell death inhibitors (10 µM ZVAD-FMK and 10 µM ferrostatin-1) for 48 h. **I** Columnar statistical chart indicated changes in the apoptosis rates of ES cell lines after inhibiting YAP1 with siRNA. **J** Detection of the intracellular ROS levels in ES cells after YAP1 knockdown with siRNA. **K** Detection of the intracellular lipid ROS levels in ES cells after YAP1 knockdown with siRNA. **L** Changes of the relative intracellular Fe2+ levels in ES cells after YAP1 inhibition. **M** WB analysis indicated changes in the apoptosis-related gene markers (PARP, Bcl2, Bax) and the ferroptosis-related marker GPX4 after YAP1 inhibition in ES cells. The corresponding original western blots are shown in Fig. S13. **N** WB analysis of the YAP1 protein expression in siAURKA ES cells treated with or without oeNPM1. The corresponding original western blots are shown in Fig. S13. **O** CoIP of YAP1 and NPM1 (WT, Thr95D, or Thr95A) from lysates of 293T cells. The corresponding original western blots are shown in Fig S13. Values represented the mean ± SD from 3 independent experiments. **p < 0.01, ***p < 0.001, ****p < 0.0001.

In this study, it was found that AURKA phosphorylated NPM1 Thr95 to promote ES apoptosis and ferroptosis resistance. Bioinformatic analysis was carried out to investigate the molecular mechanism through which NPM1 increased ES apoptosis and ferroptosis resistance. The overlap of genes interacted with NPM1 (http://www.hitpredict.org/) [22] and genes associated with ferroptosis (https://genecards.weizmann.ac.il/v3/) [23] contained 9 known oncogenes in ES, including YAP1, BRD4, MYC, MDM2, SOX2, SP1, JUN, EGFR, and PRC1(Fig. 7A). Wang et al. reported that AURKA may boost YAP1 stability in lung cancer [24], and YAP1 was reported to play a crucial role in regulating ferroptosis [25, 26], which led us to believe that AURKA may promote YAP1 stability through NPM1. The result of immunoprecipitation demonstrated that NPM1 and YAP1 were physically interacting (Fig. 7B, C). As expected, AURKA or NPM1 knockdown dramatically decreased YAP1 protein expression (Fig. 7D, E), while having no discernible effect on mRNA expression (Fig. S4A, B). Knockdown of YAP1 significantly impaired cell growth (Fig. 7F), and induced cell apoptosis and ferroptosis in ES cells (Fig. 7G–M). Consistently, WB results revealed that knockdown of AURKA significantly decreased the expression of YAP1, which was partly reversed by overexpression of NPM1(Fig. 7N). The above results made us wonder whether the phosphorylation level of NPM1 Thr95 affected the binding of NPM1 to YAP1. The results of immunoprecipitation showed that the phosphorylated status of NPM1 Thr95 is important for the binding of NPM1 to YAP1 (Fig. 7O). Altogether, these findings provided strong evidence that AURKA promotes ES cell apoptosis and ferroptosis resistance through the NPM1/YAP1 axis.

## Discussion

Following osteosarcoma, ES is the second most frequent bone or soft tissue tumor occurring in childhood and adolescence. Despite the introduction of multimodal treatment regimens, including surgery, radiotherapy, and multi-agent chemotherapy, the long-term survival rate of patients with recurrent or metastatic ES remains unacceptably low. Considering the adverse outcomes of ES, uncovering novel potential molecular mechanisms underlying ES development is highly vital for improving survival outcomes.

In this study, it was reported that AURKA inhibitor TCS7010 markedly repressed ES growth in vitro and in vivo by inducing the apoptosis and ferroptosis. As shown here, AURKA acted as an endogenous repressor of apoptosis and ferroptosis by phosphorylating NPM1 Thr95, thus stabilizing YAP1. These findings not only reveal the new molecular mechanism of AURKA in ES growth but also highlight the notion that induction of apoptosis and ferroptosis may be critical for anticancer therapies. The results indicate that AURKA may be a therapeutic target of ES.

AURKA is a member of the highly homologous family of serine/threonine kinases, playing an important role in the regulation of cell cycle and division. However, abnormally expressed AURKA may act as an oncogene in a variety of cancers [27]. The expression level and role of AURKA in ES remain elusive. In this study, the expression level of AURKA was found to be upregulated in ES, consistent with the results of previous studies that the expression level of AURKA is upregulated in numerous types of cancers, including lung cancer, breast cancer, and gastric cancer [28–30]. In this study, we noted that AURKA was associated with apoptosis and ferroptosis in ES. It made us wonder whether AURKA played a crucial role in ES. As expected, the expression of AURKA was significantly associated with the shorter OS and EFS of ES individuals. To the best of our knowledge, this is the first time to confirm that AURKA is upregulated in ES and related to ES clinically. However, the sample size needs to be enlarged to further confirm this conclusion.

After analyzing clinical samples, the experimental model was utilized to search for further support. It was found that knockdown of AURKA significantly decreased growth of ES cells in vitro and in vivo, in line with previous reports that AURKA acts as an oncogene in various cancers, including gastric cancer, rhabdomyosarcoma, and colorectal cancer [31, 32]. Furthermore, we innovatively found the apoptosis and ferroptosis regulation of AURKA in ES cells. Both genetic knockdown of AURKA and pharmacological inhibition with the AURKA inhibitor TCS7010 in ES cells caused significant apoptosis and ferroptosis. While, to further establish the impact of AURKA, we used Alisertib to inhibit AURKA in ES cells, and observed the same phenomenon (Fig. S5). Notably, the apoptosis induced by AURKA inhibition was found to be partly dependent on activating ferroptosis (Fig. S6), which indicated that the main effect of TCS7010 on ES is to induce ferroptosis.

Ommer et al. demonstrated that AURKA inhibition destabilized PAX3-FOXO1 and MYCN to induce rhabdomyosarcoma cell death. AURKA prevented the Fbxw7-mediated degradation of MYCN, which facilitated the growth of MYCN-amplified neuroblastoma cells. AURKA promoted gastric cancer cell growth by regulating GSK-3β [31, 33–35]. However, there are few studies on the ferroptosis role and mechanism of AURKA. Li et al. revealed that AURKA was a negative regulator for ferroptosis in NSCLC [36]. Zhao et al. demonstrated that AURKA was a ferroptosis-related gene associated with worse survival outcomes in ES [37], but they did not further explore the specific mechanisms by which AURKA regulated ferroptosis in ES. For all we know, this is the first time to report that AURKA promotes apoptosis and ferroptosis resistance of ES cells *via* phosphorylating NPM1 Thr95.

NPM1 has been reported to be upregulated in various human cancers, including breast cancer, colorectal cancer, lung cancer, and ES, which promotes cell proliferation and apoptosis resistance [38–41]. Zhou et al. demonstrated that NPM1 decreases ES cell apoptosis, consistent with our study [41]. We are the first to report the inhibitory effect of NPM1 on ferroptosis in ES. For the mechanism, we found that NPM1 was physically connected with YAP1, and the phosphorylated status of NPM1 Thr95 was important for the binding of NPM1 to YAP1. In this study, it was innovatively demonstrated that AURKA promoted ES apoptosis and ferroptosis resistance through the NPM1/YAP1 axis.

Inevitably, there were also some limitations. First, the exact mechanisms by which NPM1 stabilized YAP1 remained unclear. The detailed mechanism needs to be further studied. Second, the sample size for survival analysis was relatively small, which might cause some bias. Third, the animal models used in this study were cell line-derived xenografts, so the conclusions need to be further validated by patient-derived xenografts.

## Conclusions

In summary, the expression level of AURKA is upregulated in ES, and AURKA inhibition induces the apoptosis and ferroptosis in ES cells. As for the fundamental mechanism, AURKA facilitates anti-apoptosis and anti-ferroptosis capabilities in ES through the NPM1/YAP1 axis. Therefore, AURKA may be a potential target for the treatment of ES.

## Materials and methods

### Antibodies and reagents

The antibodies to PARP (66520-1-Ig), Ki67 (27309-1-AP), NPM1 (10306-1-AP), and GPX4 (67763-1-Ig) were purchased from Proteintech. The antibodies to GPX4 (A1933), GAPDH (AC001), p-NPM1 Thr199 (AP0836), Bax (A19684), Bcl2 (A0208), and YAP1 (A1002) were purchased from ABclonal. The antibody to AURKA (#14475) was purchased from Cell Signaling Technology. The antibodies to p-NPM1 Ser125 (AF3740), p-NPM1 Ser4 (AF8496), and p-NPM1 Thr95 (AF2372) were purchased from Affinity Biosciences. The small-molecule library, TCS7010, ZVAD-FMK, necrostatin-1, ferrostatin-1, and chloroquine were purchased from TargetMol.

### Patient tissues and characteristics

Tissue paraffin sections were collected from the tissue bank at the Sun Yat-sen Memorial Hospital of Sun Yat-sen University. The patients enrolled were pathologically diagnosed at our center from May 2002 to November 2020. The inclusion criteria were as follows: (1) patients aged ≤18 years, (2) those pathologically diagnosed with ES, (3) those with complete medical records and follow-up data. This study was approved by the Sun Yat-sen Memorial Hospital of Sun Yat-sen University Institutional Review Board and the Research Ethics Committee.

### Immunohistochemistry (IHC) assay

The sections were deparaffinized in xylene and rehydrated with gradient concentrations of alcohol, followed by treatment with 3% H_2_O_2_ for 10 mins to block endogenous peroxidase activity. Then the sections were blocked by 10% goat serum for 30 mins at 37 °C and were immunostained with primary antibodies (anti-human AURKA, Ki67 and GPX4) at 4 °C overnight. The next day, the sections were incubated with goat anti-mouse/rabbit secondary antibody for 30 mins at 37 °C. The expression level of protein was semi-quantitatively detected based on staining intensity and distribution, using the immunoreactive score as described elsewhere. The sections were split into low expression whose score ranged from 0 to 4 points, and high expression whose score ranged from 5 to 9 points.

### Cell culture

The human ES cell lines (A673, RDES, SKNMC, and SK-NEP-1 cells) and the human bone marrow mesenchymal stem cell (BMSC) were obtained from Cobioer Biosciences Co., Ltd. (Nanjing, China) and cultured with a complete medium supplemented with 10% or 15% FBS as recommended by the manufacturer. These cells were then incubated in a 5% CO_2_ humidified incubator at 37 °C. All cell lines had been authenticated and free from mycoplasma.

### High-throughput drug library screening

The commercially available small molecule compound library including 294 compounds was purchased from TargetMol. The ES cell line A673 was used to perform the high-throughput drug library screening in vitro. The cells were inoculated in 96-well plates at a density of 4000 cells/well and incubated with each drug at a working concentration of 2 μM for 72 h. Then cell viability was detected with the CCK-8 reagent at 450 nm using a microplate reader. Drugs achieving a cell survival rate of less than 40% were defined as effective drugs.

### Quantification of intracellular ROS level

The levels of intracellular ROS were detected with a flow cytometer using DCFH-DA (Beyotime, S0033S) as the fluorescent probe according to the manufacturer’s protocol. In brief, ES cells were incubated with 10 μM of DCFH-DA for 20 mins at 37 °C. Subsequently, the cells were washed for 3 times with a FBS-free medium and detected by flow cytometry.

### Lipid ROS assay

The BODIPY 581/591 C11 undecanoic acid (Thermo Fisher Scientific, D3861) was used to assess the relative lipid ROS level in cells. BODIPY 581/591 C11 were added into complete medium and incubated at 37 °C for 30 mins. Subsequently, the cells were harvested, washed twice with precooled PBS, and detected by flow cytometry.

### Iron assay

The total levels of intracellular iron were investigated with the Iron Assay Kit (Solarbio, BC5415) following the manufacturer’s protocol. The cells were rapidly homogenized in Regent I, sonicated on ice for 5 mins, and centrifuged at 10,000 × *g* at 4 °C for 10 mins. Then 200 μL of samples together with 100 μL of Regent II were added into a 1.5 mL EP tube, fully mixed, and incubated for 10 mins at 37 °C. 100 μL of CHCL3 was added into the samples, shaken for 5 mins and then centrifuged at 12000 × *g* for 10 mins at room temperature. A total of 200 μL of cell samples and diluted standard samples were added into 96-well plates, and then the absorbance was measured with a microplate reader at 593 nm. The corresponding iron concentration in each sample was calculated based on the standard curves.

### Tunel staining

The sections were deparaffinized in xylene rehydrated with gradient concentrations of alcohol and permeabilized in 0.3% Triton‐X 100 for 20 mins. Tunel assays were carried out according to the manufacturer’s instructions (Procell, China). Briefly, the cells were first incubated in a terminal dexynucleotidyl transferase (TdT) reaction cocktail for 1 h at 37 °C, then the nucleus was stained with DAPI and photographed with confocal microscope.

### RNA isolation and quantitative reverse transcription-polymerase chain reaction (RT-qPCR)

Total RNA was extracted from cultured cells with TRIzol reagent (TaKaRa Bio Inc., Kusatsu, Japan), and reversely transcribed into cDNA using a Reverse Transcriptase Kit (Invitrogen, Carlsbad, CA, USA), followed by RT-qPCR using SYBR Premix Ex Taq II (Takara, Shiga, Japan) in an ABI-7300 Real-Time PCR system (Applied Biosystems, Foster City, CA, USA), with glyceraldehyde-3-phosphate dehydrogenase (GAPDH) as an internal control. Three replicates of each sample were amplified in a 10 μL qPCR mixture using the iTaq Universal SYBR Green One-step Kit (Bio-Rad Laboratories, Inc., Hercules, CA, USA). The sequences of primers for RT-qPCR are listed in Table S1.

### Cell transfection and viral infection

For the transient knockdown assay, the small interfering RNAs (siRNAs), AURKA siRNA (siAURKA), NPM1 siRNA (siNPM1), YAP1 siRNA (siYAP1), and synthetic sequence-scrambled siRNA (siNC) were obtained from GenePharma (Shanghai, China). The short hairpin RNAs (shRNAs) of AURKA, NPM1, and YAP1 were purchased from GeneCopoeia (Guangzhou, China). The transfection and infection procedures were conducted following the manufacturer’s instructions. Stable cell lines were selected using the appropriate antibiotics for at least 48 h after transfection. The efficiency verification of siRNA, shRNA, and plasmid is in Fig. S2.

### Colony formation assay

To detect the effect of genes on cell growth, 8 × 10^3^ cells (A673 and RDES cells) transfected with shNC or corresponding shRNA were inoculated into 6-well plates. After incubation for 10 days at 37 °C and 5% CO_2_, the cells were fixed with 4% paraformaldehyde, stained with 0.1% crystal violet, and counted.

### Apoptosis assay

Cell apoptosis assay was performed with Annexin V-Alexa Fluor 647/7-AAD Apoptosis Detection Kit (4 A Biotech Co., Ltd., Beijing, China) following the manufacturer’s instructions. In brief, 4 × 10^5^ cells were harvested, washed twice with precooled PBS, and resuspended in 400 μL of binding buffer. Afterward, the cells were treated with 5 μL of Annexin V-Alexa Fluor 647 and 7-AAD for 5 mins away from light, followed by flow cytometry (SP6800, Sony, Japan) within 1 h.

### Western blotting (WB)

The cells were washed with precooled PBS and lysed for 30 mins on ice in RIPA lysis buffer containing protease inhibitor cocktail (KeyGene, Shanghai, China). Subsequently, colorimetric detection and quantitation of total protein were conducted using a bicinchoninic acid (BCA) assay kit (Thermo Fisher Scientific, Waltham, MA, USA). Proteins were separated by sodium dodecyl sulfate-polyacrylamide gel electrophoresis (SDS-PAGE), transferred onto a polyvinylidene difluoride (PVDF) membrane (Sigma-Aldrich), and blocked with 5% skimmed milk for 1 h at room temperature. Then WB was performed with primary antibodies, including rabbit anti-human AURKA, rabbit anti-human NPM1, mouse anti-human YAP1, rabbit anti-human p-NPM1 Ser125, rabbit anti-human p-NPM1 Thr199, rabbit anti-human p-NPM1 Ser4, rabbit anti-human p-NPM1 Thr95, rabbit anti-human actin, and mouse anti-human GAPDH.

### Co-immunoprecipitation (Co-IP) assay

For the Co-IP assay, the cells were lysed in 50 mM Tris-HCl (pH7.4), 150 mM NaCl, 1 mM EDTA, 1% NP-40, 1 mM DTT, and protease inhibitor cocktail. Then, 1 mg of the cell lysates were incubated with the corresponding AURKA/NPM1 antibodies overnight at 4 °C, with homologous immunoglobulin G (IgG) as a negative control. Finally, each lysate was added with an equivalent amount of beads (Thermo Fisher Scientific) and incubated overnight at 4 °C. Next, beads were washed with lysis buffer 3 times. Reactions were subjected to WB analysis. Whole-cell lysates served as an input control, and normal IgG acted as a negative control.

Proteins were separated by sodium dodecyl sulfate-polyacrylamide gel electrophoresis (SDS-PAGE), then the gel was silver stained. After that, the different bands were cut for mass spectrometry analysis.

### In vivo xenograft mouse models

To analyze the effect of AURKA on tumor growth in ES, 3–4-week-old female nude mice were injected subcutaneously with 4 × 10^6^ A673 cells via the right flank. The tumor volume (mm^3^) was calculated as follows: 0.5 × D1 × D2 × D2, where D1 and D2 are the largest diameter and the smallest diameter of a given tumor, respectively. The growth and volume of the tumor were monitored every 3 days for up to 4 weeks using a caliper. All the mice were euthanized after 4 weeks of inoculation.

### Statistical analysis

Data analysis was carried out using GraphPad Prism 8 or SPSS software (version 22). Continuous variables were presented as mean ± standard (SD) deviation, and differences were compared using Student’s *t*-test or one-way analysis of variance. The differences in categorical variables were compared using the χ^2^ test. Survival rates were calculated by the Kaplan-Meier method and the comparisons were performed using Log-rank test. All tests were two-sided, and *p* < 0.05 was considered a significant difference.

### Supplementary information

Supplementary materials

Original Data File

## Data Availability

The original contributions presented in the study are included in the article/supplementary materials. The authenticity of this article has been validated by uploading the key raw data to the Research Data Deposit (RDD) public platform (https://rdd.sysu.edu.cn/), with the approval RDD number as RDDYJ862009. Further inquiries can be directed to the corresponding authors.
