# Supplementary material for: AURKA inhibition induces Ewing’s sarcoma apoptosis and ferroptosis through NPM1/YAP1 axis
Source: Cell Death Dis. 2024 Jan 29;15(1):99. doi: 10.1038/s41419-024-06485-0 (PMC10825207; doi:10.1038/s41419-024-06485-0)
Supplement: Supplementary file 1 — Supplementary materials [file 41419_2024_6485_MOESM1_ESM.pdf]

Table S1 The sequence of primers used in this study

Name	Forward primer (5'->3')	Reverse primer (5'->3')
GAPDH	TCGTCATGGGTGTGAACCAT	TGATGATCTTGAGGCTGTTGTCA
AURKA	AATCTGGAGGCAAGGTTCGA	GCTTCGCCAACCCAATAAGT
NPM1	GTCCGCCTTCTCTCCTACCT	CATTGCCTCTGCTTCAACAA
YAP1	GTGGCACCTATCACTCTCGA	TGGCTTCAAGGTAGTCTGGG

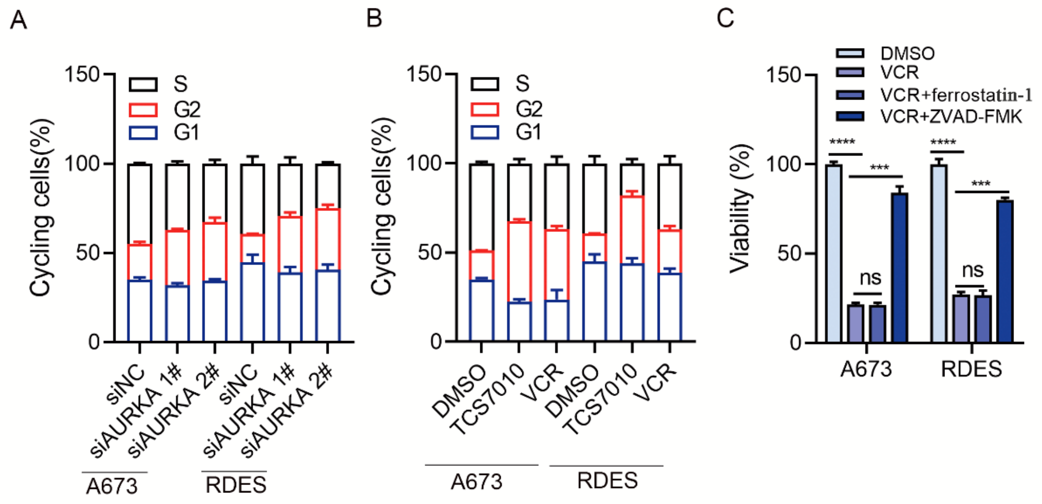

Fig S1. AURKA inhibited ferroptosis in a cell cycle-independent manner.

(A) Cell cycle analysis in AURKA knockdown cells. (B) Cell cycle analysis in A673 and RDES cells following TCS7010 (1μM) and VCR (4nM) for 24h. (C) ES cells were treated with VCR (4nM) in the absence or presence of indicated cell death inhibitors for 48h, and then cell viability was assayed. ***p<0.001, ****p< 0.0001.

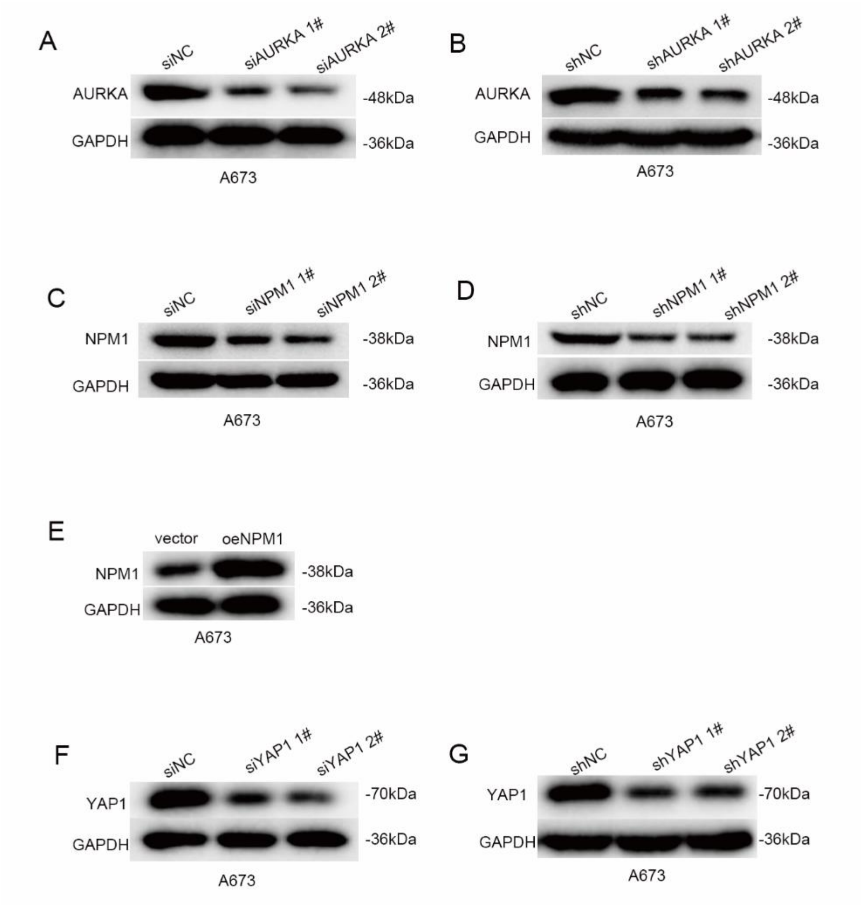

Fig S2. Efficiency of siRNA, shRNA and plasmid.

(A) WB analysis of the efficiency of siAURKA in A673. (B) WB analysis of the efficiency of shAURKA in A673. (C) WB analysis of the efficiency of siNPM1 in A673. (D) WB analysis of the efficiency of shNPM1 in A673. (E) WB analysis of the efficiency of the NPM1 overexpressed plasmid in A673. (F) WB analysis of the efficiency of siYAP1 in A673. (G) WB analysis of the efficiency of shYAP1 in A673. The corresponding original western blots were showed in Fig. S14.

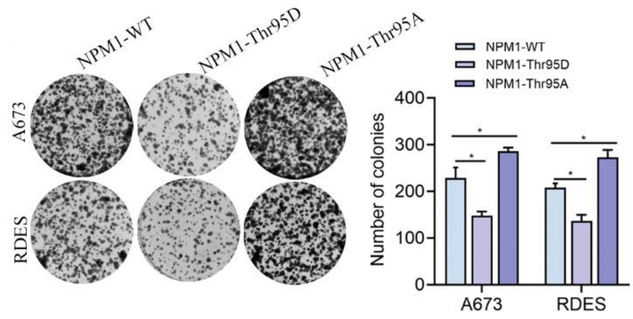

Fig S3. The phosphorylated status of NPM1 Thr95 was important for ES cell growth. Values represented the mean±SD from 3 independent experiments. *p<0.05.

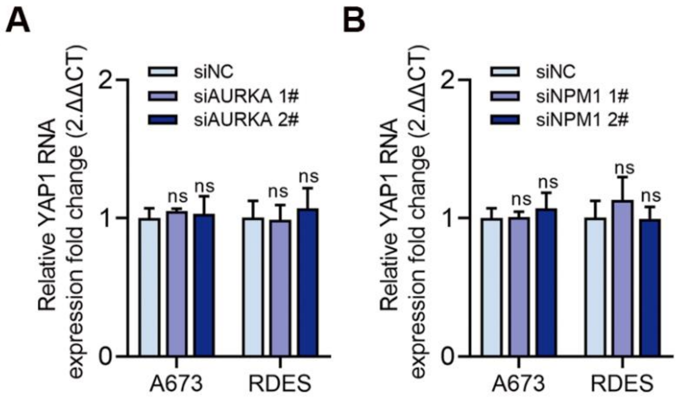

Fig S4. The mRNA levels of YAP1 after AURKA or NPM1 knockdown.

(A) The mRNA level of YAP1 after AURKA knockdown. (B) The mRNA level of YAP1 after NPM1 knockdown. Values represented the mean±SD from 3 independent experiments. ns: non significance.

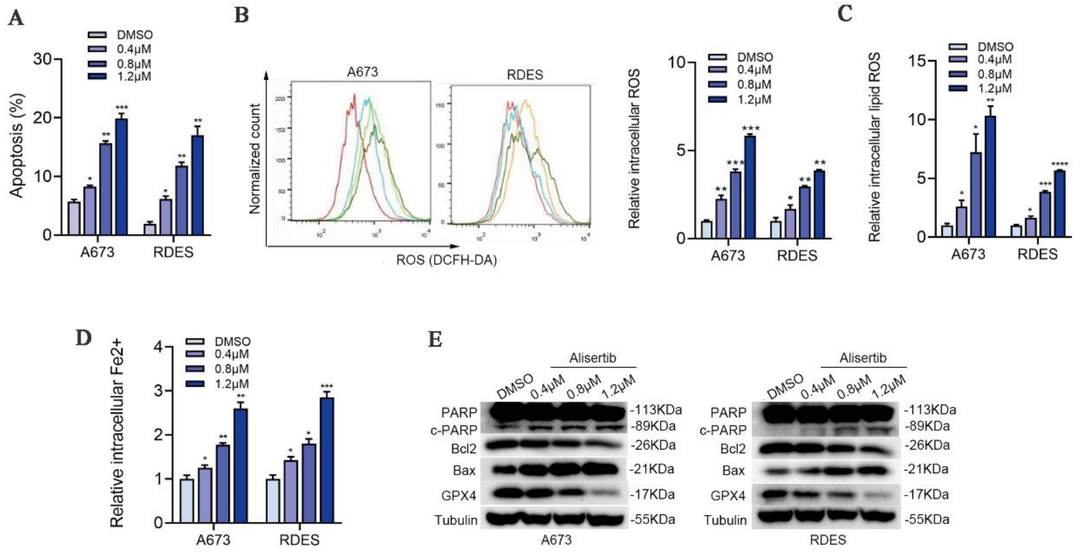

Fig S5. AURKA inhibition with Alisertib induced apoptosis and ferroptosis in ES cells.

(A) Columnar statistical chart indicated changes in the apoptosis rates of ES cell lines after AURKA inhibition with Alisertib. (B) Detection of the intracellular ROS levels in ES cells after Alisertib treatment. (C) Detection of the intracellular lipid ROS levels in ES cells after Alisertib treatment. (D) Changes of the relative intracellular Fe2+ levels in ES cells after AURKA inhibition with Alisertib. (E) WB analysis indicated changes of the apoptosis-related gene markers (PARP, Bcl2, Bax) and the ferroptosis-related marker GPX4 after AURKA inhibition with Alisertib in ES cells. The corresponding original western blots were showed in Fig. S15. Values represented the mean±SD from 3 independent experiments. *p<0.05, **p<0.01, ***p<0.001, ****p<0.0001.

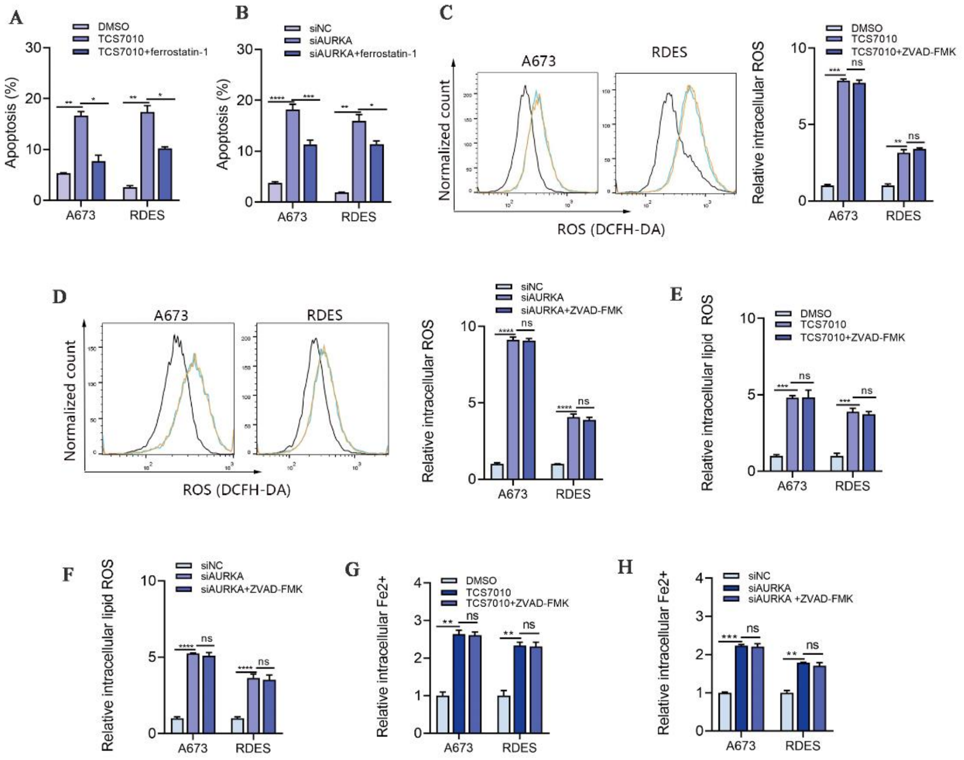

Fig S6. The apoptosis induced by AURKA inhibition was found to be partly dependent on activating ferroptosis.

(A, B) Columnar statistical chart indicated changes in the apoptosis rates of ES cell lines with AURKA inhibition (treated with TCS7010 or siAURKA) alone or in combination with ferroptosis inhibitor ferrostatin-1. (C, D) Detection of the intracellular ROS levels in ES cells with AURKA inhibition (treated with TCS7010 or siAURKA) alone or in combination with apoptosis inhibitor ZVAD-FMK. (E, F) Detection of the intracellular lipid ROS levels in ES cells with AURKA inhibition (treated with TCS7010 or siAURKA) alone or in combination with apoptosis inhibitor ZVAD-FMK. (G, H) Changes of the relative intracellular Fe2+ levels in ES cells with AURKA inhibition (treated with TCS7010 or siAURKA) alone or in combination with apoptosis inhibitor ZVAD-FMK.
