# Supplementary material for: AURKA inhibition induces Ewing’s sarcoma apoptosis and ferroptosis through NPM1/YAP1 axis
Source: Cell Death Dis. 2024 Jan 29;15(1):99. doi: 10.1038/s41419-024-06485-0 (PMC10825207; doi:10.1038/s41419-024-06485-0)

Fig S7. Original full length western blots for Fig. 2B.

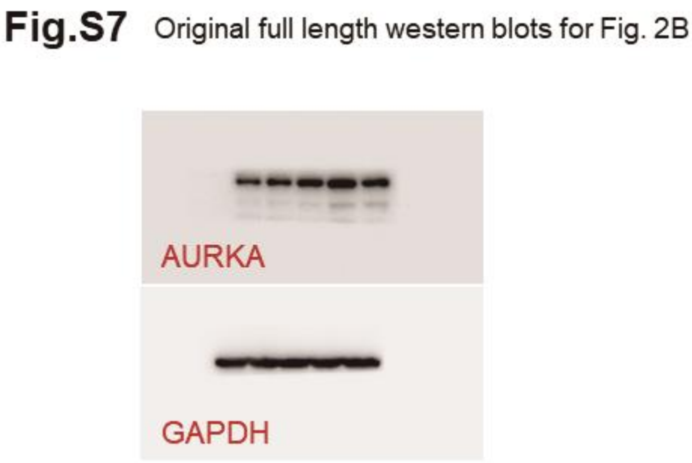

Fig S8. Original full length western blots for Fig. 3J.

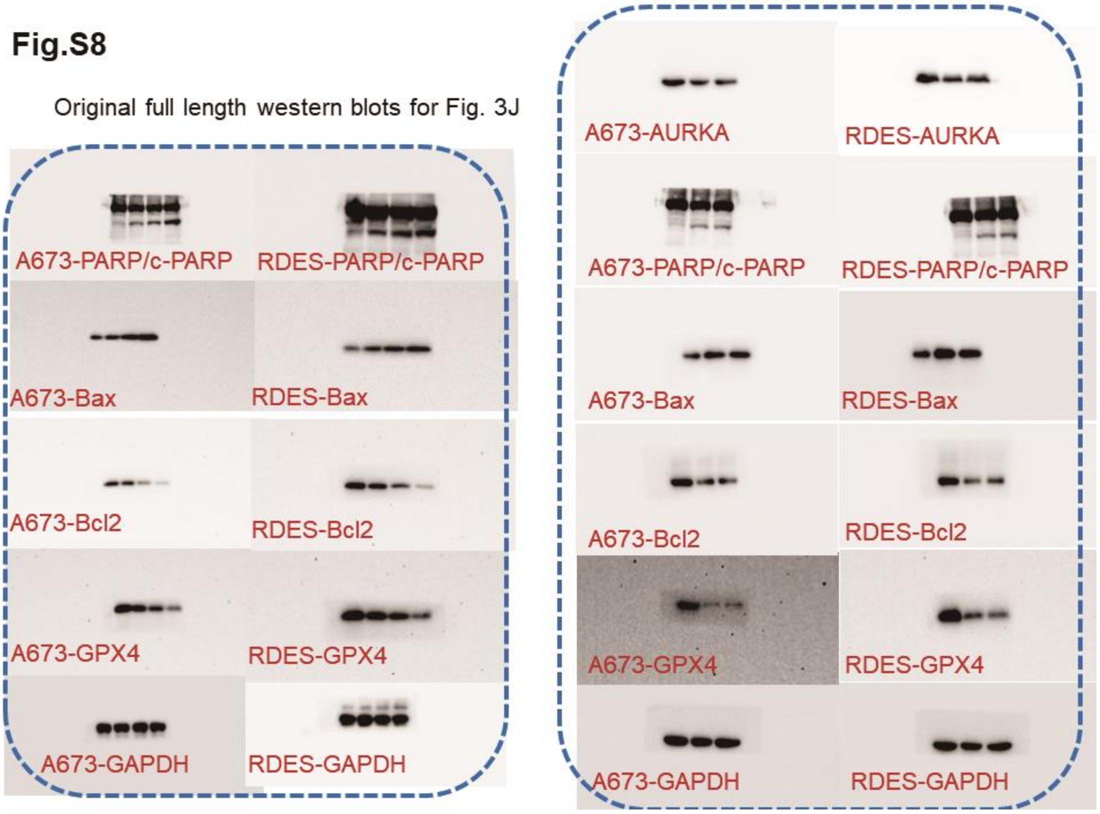

Fig S9. Original full length western blots for Fig. 5C.

Fig.S9

Original full length western blots for Fig. 5C

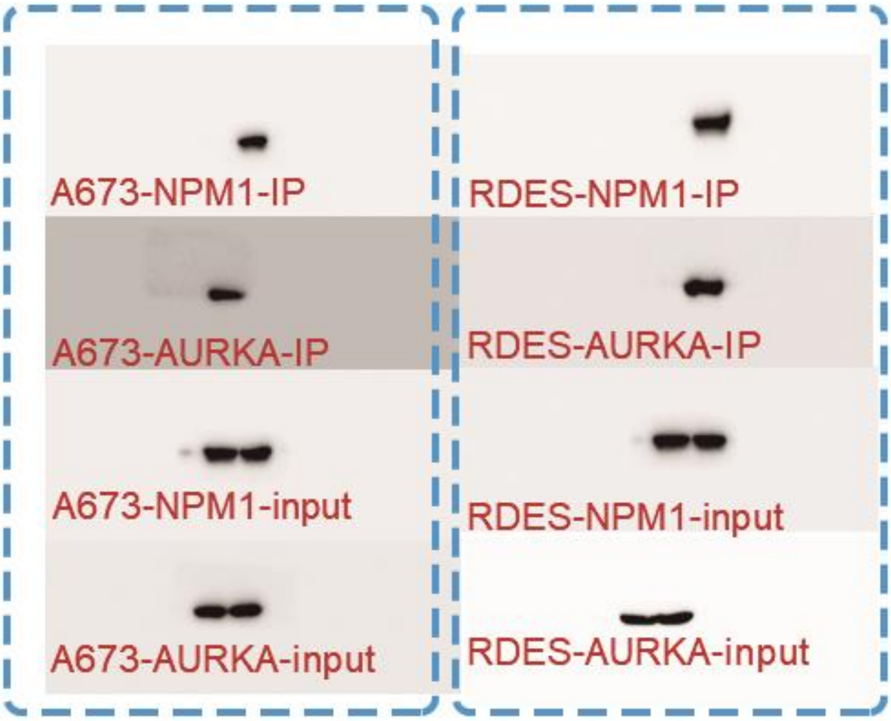

Fig S10. Original full length western blots for Fig. 5D and Fig. 5E.

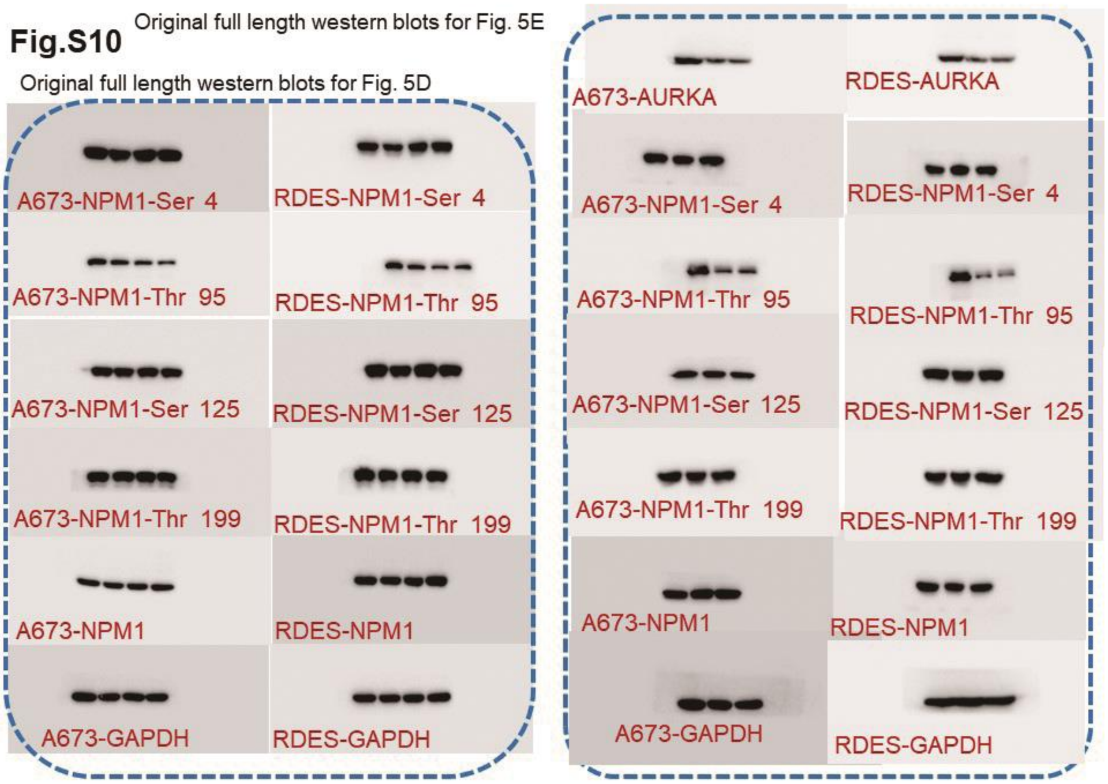

Fig S11. Original full length western blots for Fig. 5M, Fig. 7B and Fig. 7C.

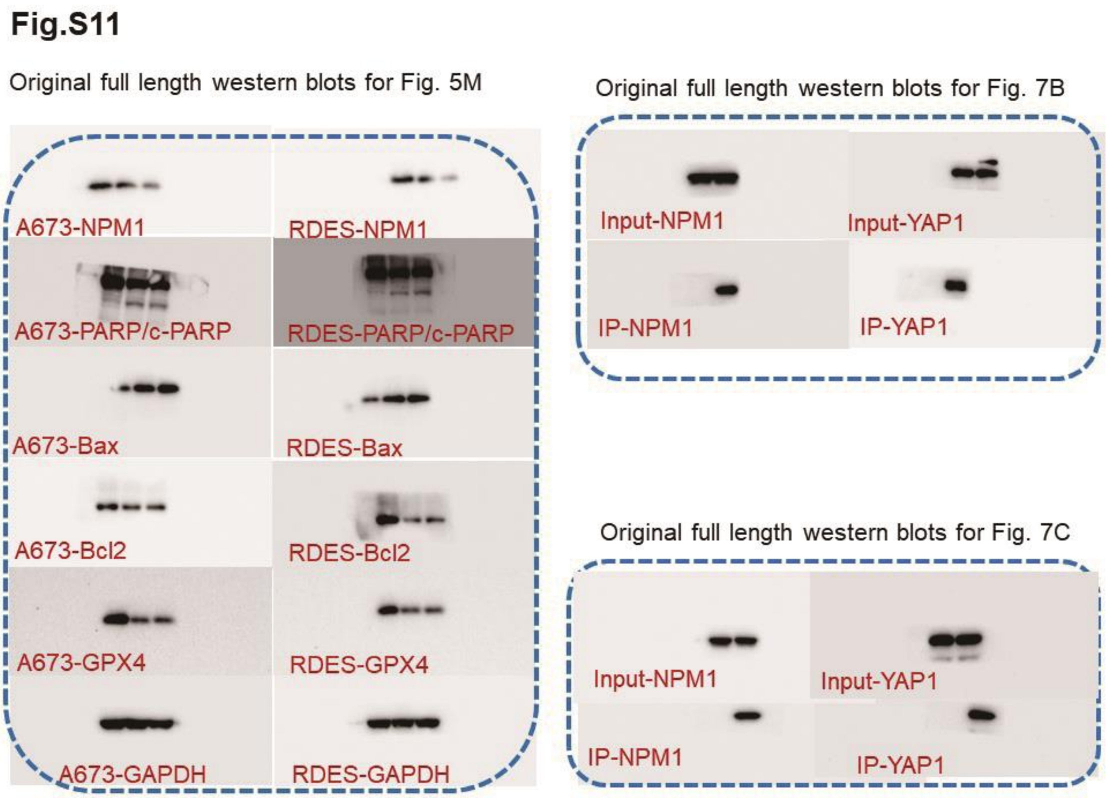

Fig S12. Original full length western blots for Fig. 7D and Fig. 7E.

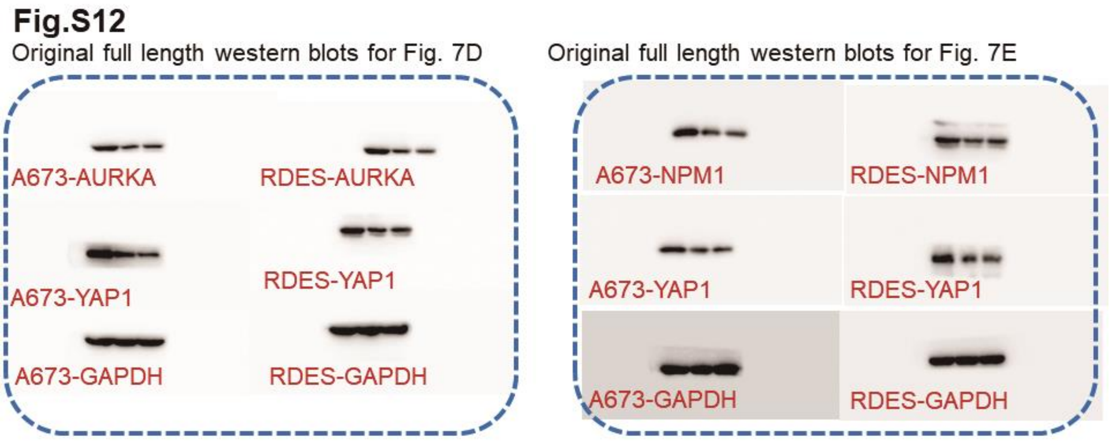

Fig S13. Original full length western blots for Fig. 7M, Fig. 7N and Fig. 7O.

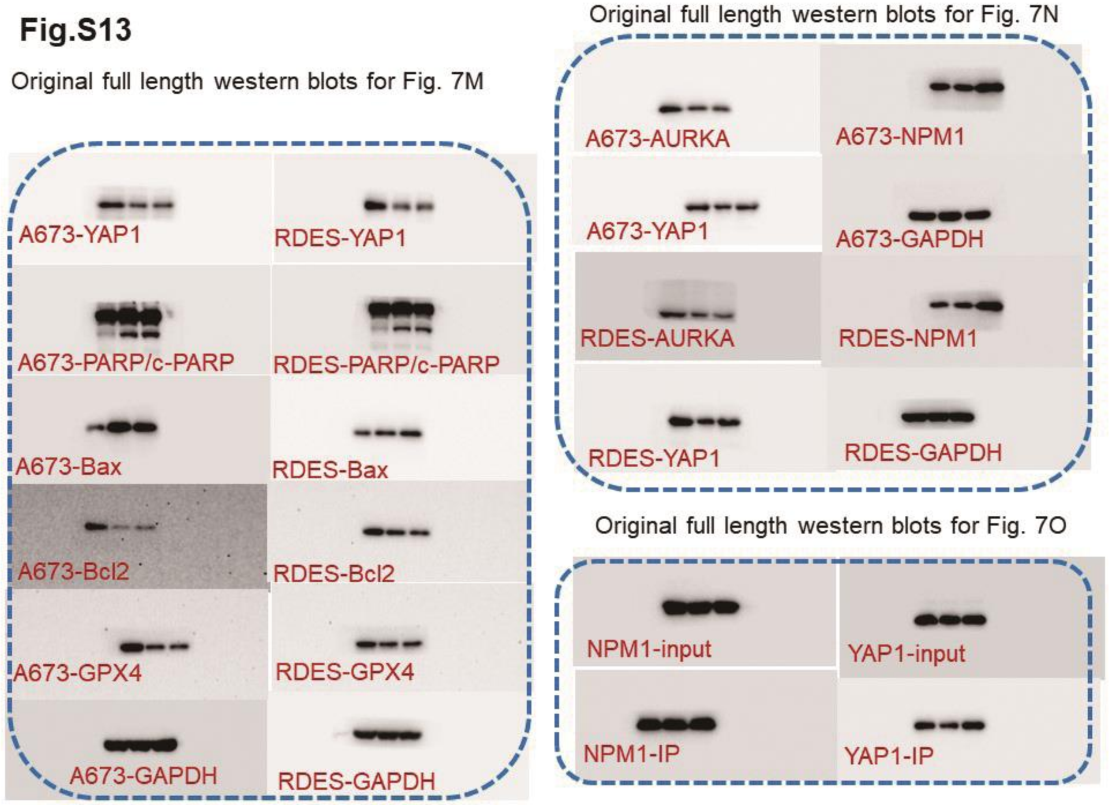

Fig S14. Original full length western blots for Fig.S2.

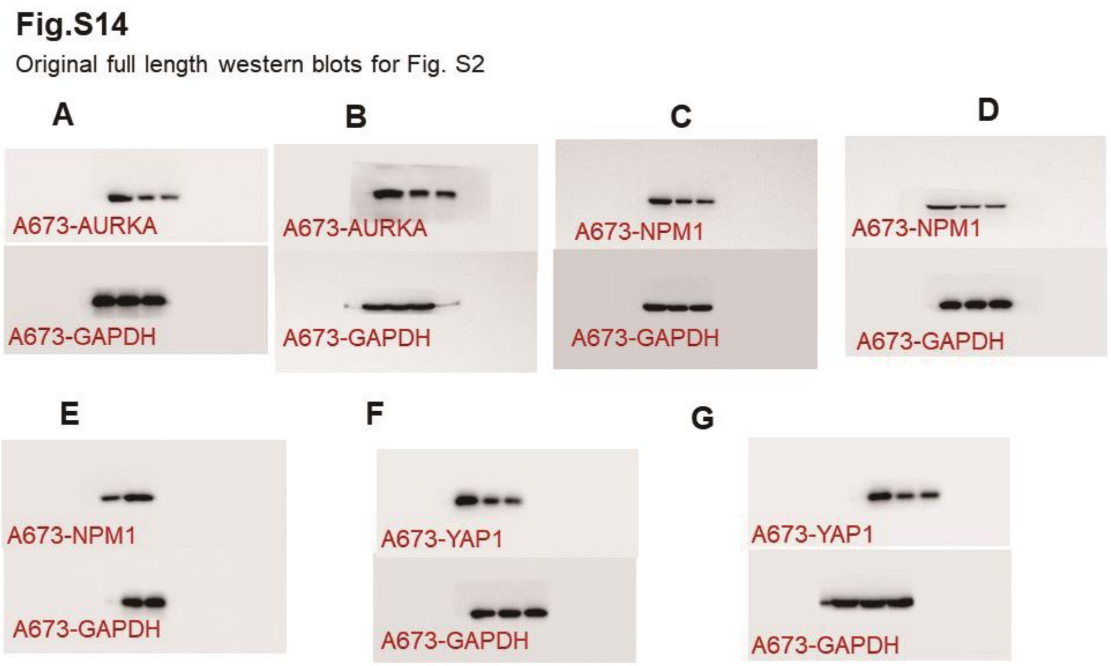

Fig S15. Original full length western blots for Fig.S5.

Fig.S15 Original full length western blots for Fig.S5

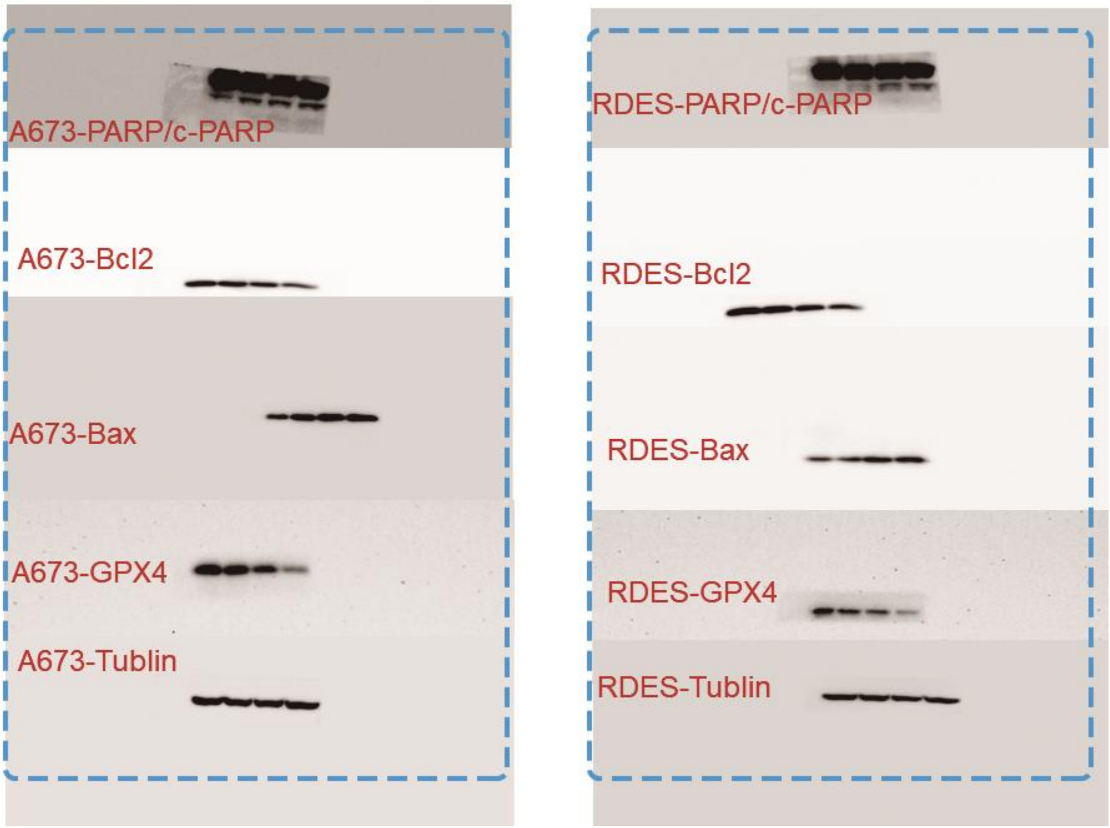

Supplement: Supplementary file 2 — Original Data File [file 41419_2024_6485_MOESM2_ESM.pdf]
